# Optical Control of Tumor Induction in the Zebrafish

**DOI:** 10.1038/s41598-017-09697-x

**Published:** 2017-08-23

**Authors:** Zhiping Feng, Suzy Nam, Fatima Hamouri, Isabelle Aujard, Bertrand Ducos, Sophie Vriz, Michel Volovitch, Ludovic Jullien, Shuo Lin, Shimon Weiss, David Bensimon

**Affiliations:** 1 Department of Molecular, Cellular and Integrative Physiology, University of California at Los Angeles, Los Angeles, California, USA; 2 Department of Ecology and Evolutionary Biology, University of California at Los Angeles, Los Angeles, California, USA; 3 Laboratoire de Physique Statistique, Ecole Normale Supérieure, PSL Research University, Paris, France; 4 IBENS, CNRS-UMR8197, INSERM-U1024, PSL Research University, Paris, France; 5 École Normale Supérieure, PSL Research University, UPMC Univ Paris 06, CNRS, Département de Chimie, PASTEUR, Paris, France; 6 Sorbonne Universités, UPMC Univ Paris 06, ENS, CNRS, PASTEUR, Paris, France; 7 Center for Interdisciplinary Research in Biology (CIRB), College de France, and CNRS UMR 7241, and INSERM U1050, Paris, France; 8 Department of Life Sciences, Paris-Diderot University, Sorbonne-Paris-Cité, Paris, France; 9 Department of Biology, Ecole Normale Supérieure, PSL Research University, Paris, France; 10 Department of Molecular, Cell and Developmental Biology, University of California at Los Angeles, Los Angeles, California, USA; 11 Department of Chemistry and Biochemistry, University of California at Los Angeles, Los Angeles, California, USA; 12 Department of Chemical and Systems Biology, Present Address: Stanford University, Stanford, California, USA

## Abstract

The zebrafish has become an increasingly popular and valuable cancer model over the past few decades. While most zebrafish cancer models are generated by expressing mammalian oncogenes under tissue-specific promoters, here we describe a method that allows for the precise optical control of oncogene expression in live zebrafish. We utilize this technique to transiently or constitutively activate a typical human oncogene, *kRASG12V*, in zebrafish embryos and investigate the developmental and tumorigenic phenotypes. We demonstrate the spatiotemporal control of oncogene expression in live zebrafish, and characterize the different tumorigenic probabilities when *kRASG12V* is expressed transiently or constitutively at different developmental stages. Moreover, we show that light can be used to activate oncogene expression in selected tissues and single cells without tissue-specific promoters. Our work presents a novel approach to initiate and study cancer in zebrafish, and the high spatiotemporal resolution of this method makes it a valuable tool for studying cancer initiation from single cells.

## Introduction

For the past 60 years, zebrafish have been widely used as an important vertebrate model organism. Approximately 70% of human genes have at least one zebrafish ortholog, including 84% of the genes associated with human diseases^1^. In addition, many other advantages such as high fecundity, rapid extra-uterine development, larval transparency, inexpensive husbandry, and scalability make zebrafish a very attractive animal model^2^. Zebrafish models have provided valuable insights into pathways that underlie human disease and contributed to therapeutic discovery^2–4^. In cancer research, zebrafish have also become a popular animal model^5, 6^, with multiple transgenic lines generated that faithfully recapitulate human cancers including leukemia^7^, melanoma^8^, rhabodomyosarcoma^9^, liver cancer^10, 11^, pancreatic cancer^12^ and others^13–16^. Most of these models utilize tissue-specific promoters to drive the expression of mammalian oncogenes, and they therefore have limited spatial and/or temporal resolution, even when paired with chemical inducers^10, 17^. Moreover, specific promoters are not yet available for certain organs and tissues, hindering investigations of tumorigenesis in those tissues.

**Figure 1:**
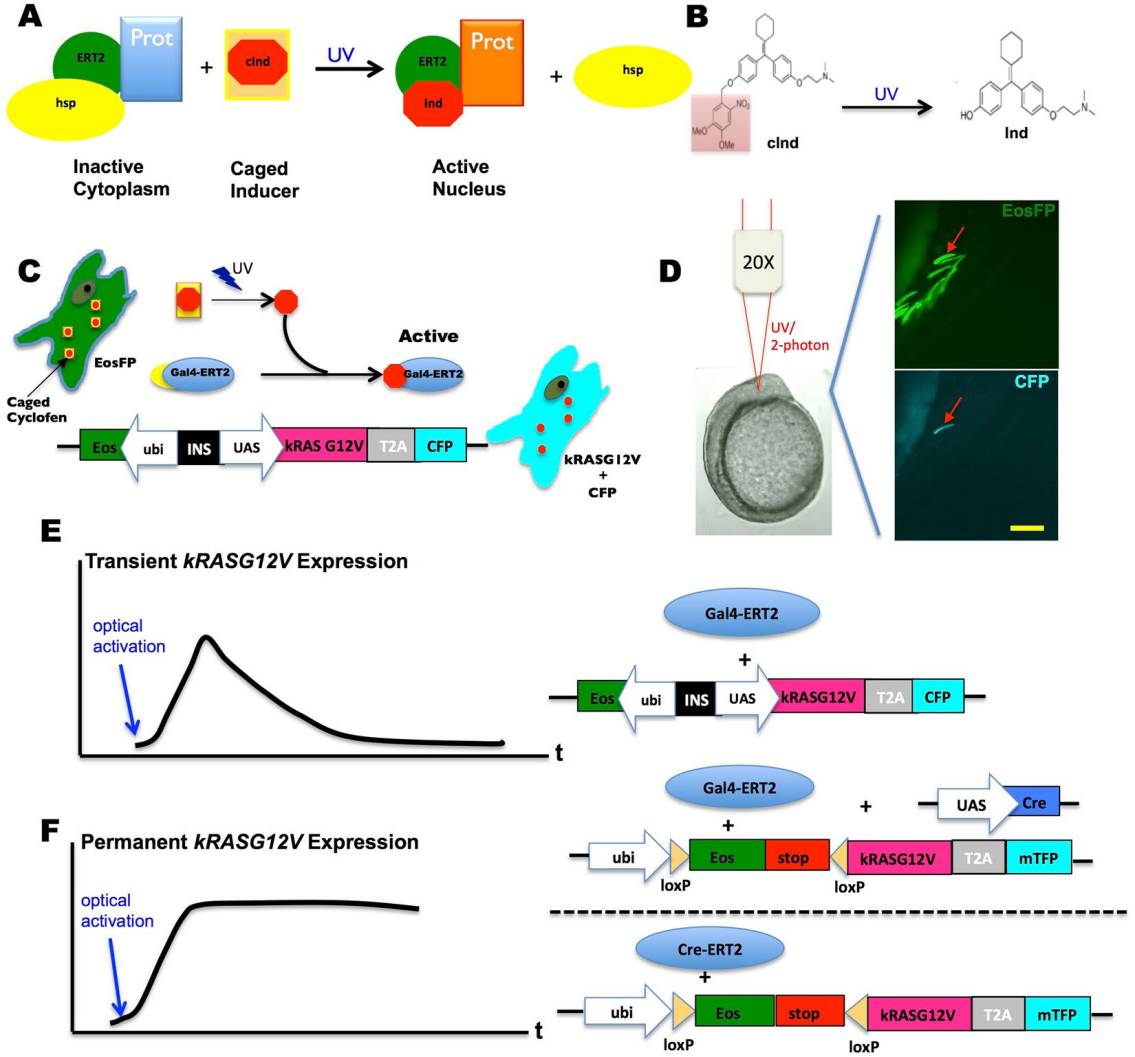
Schematic principle and system design of photo-control over specific oncogene expression. (**A**) A protein (Prot) of interest genetically fused with the estrogen receptor (ERT2) is inactivated by the complex it forms with cytoplasmic chaperones (hsp). This complex can be dissociated by binding of a specific inducer (e.g. cyclofen) to ERT2. (**B**) A caged inducer (caged cyclofen, cInd) is chemically synthesized and can be uncaged by illumination at ~370 nm (~740 nm with a two-photon source). (**C**) The scheme describes how the expression of an oncogene can be photo-activated in a live cell transfected with dual fluorescent markers (EosFP and CFP). (**D**) This example shows that expression of the oncogene was induced selectively in one muscle fiber cell through localized uncaging of cInd in a developing zebrafish embryo. The injected embryo mosaically expressed EosFP, but only the light-activated cell expressed CFP (arrow denoted). (**E,F**) Two systems were used to control the transient or constitutive expression of *kRASG12V*. Scale bar: 200 μm.

The initiation of cancer is a rare event, taking place at the level of individual transformed cells^18–20^, and only a subset of these transformed cells eventually lead to tumorigenesis^21^. In a recent study of a melanoma zebrafish model, it was reported that among all the transformed cells expressing human *B-RAFV600E*, only those expressing *crestin*, a gene usually expressed in neural crest progenitors, were able to give rise to melanoma^22^. However, why and how the crestin expression was triggered in specific transformed cells has yet to be determined. Most likely, various molecular pathways are responsible for the oncogenic transformation of different cell types, and understanding these early steps will require technologies that can target these initiating transformed cells in live organisms. An ability to control cancer induction in a spatiotemporally precise manner (i.e., in single cells of different tissues at different times) would thus significantly advance our understanding of tumorigenesis. To address these challenges, we developed a novel approach that allows us to optically control genetic expression with high spatio-temporal resolution. This approach does not require tissue-specific promoters and long exposures to possibly detrimental illumination. It relies on:

(i) the ERT2 modified ligand-binding domain of the estrogen receptor^23^ allowing nuclear translocation of fused protein such as a Cre-recombinase or a Gal4 transcriptional activator upon tamoxifen addition, and
(ii) caged cyclofen, a photo-stable analog of tamoxifen which can be photoactivated by short (few seconds) UV or two-photon illumination^24, 25^ as shown in Fig. 1A & B.

We have used this approach to induce tumors in zebrafish embryos by photo-inducing the expression of human *kRASG12V in vivo*, either transiently (with a Gal4-ERT2/UAS system) or constitutively (with a Cre-ERT2/loxP system). We demonstrate that this optogenetic technique enables rapid *kRASG12V* activation in specific regions and single cells without restriction to a specific cell type, while simultaneously fluorescently labelling these oncogene-expressing cells. We emphasize that the method developed here is suitable for spatially controlled light activation of any gene of interest.

Using this approach, we find that while transient *kRASG12V* expression during early embryogenesis (first few hours post fertilization, hpf) disrupts tissue patterning and promotes tumorigenesis, transient *kRASG12V* expression at later stages (1–2 days post fertilization, dpf) does not give rise to tumors. Periodic *kRASG12V* expression in juvenile zebrafish can lead to tumor, albeit at a very low frequency. On the other hand, we find that constitutive *kRASG12V* expression increases the probability of tumorigenesis during larval development, and may result in tumor formation in the adult fish. By analysing the correlation of tumorigenesis probability and the number of *kRASG12V* expressing cells, we deduced that the probability of tumorigenesis due to a single transformed cell at 3dpf is about 0.5% in this specific experimental context. Our approach therefore provides experimental access to the very early stages of cancer initiation and could facilitate new strategies for cancer prevention, early diagnosis and treatment.

## Results

### Design of optically controlled expression of a *kRASG12V* oncogene in live zebrafish embryos

The main motivation of this work is to develop an optical method that allows for cancer induction in a live animal, at a high spatiotemporal resolution. Our approach involves fusing a specific protein to the modified ligand binding domain of human estrogen receptor (ERT2)^23^. As shown in Fig. 1A, such a fusion protein is sequestered in the cytoplasm by the complex it forms with chaperones. The fused protein can be released from this complex upon binding of a ligand (e.g. tamoxifen) to the ERT2 domain. We have shown that cyclofen^24, 25^, a photo-stable analog of tamoxifen, has similar affinity to the ERT2 domain, and can be chemically caged and uncaged (i.e. photo-activated) upon UV (375 nm) or two-photon (750 nm) illumination (Fig. 1B). Binding of (uncaged) cyclofen to the ERT2 domain subsequently releases the fused protein from its chaperone complex, thereby effectively turning on its activity. Here, we use this system to optically turn on or off the expression of the human oncogene, *kRASG12V*. We engineered a Gal4-ERT2/UAS system to photo-activate the expression of *kRASG12V* and a co-expressed cyan fluorescent protein marker, CFP or mTFP (Fig. 1C). Figure 1D demonstrated the transient optical activation of *kRASG12V* in a single cell of a transgenic mosaic embryo upon 1 minute local UV illumination at 10 hpf. To test whether transient or constitutive expression of *kRASG12V* could induce tumorigenesis, we created two systems to optically control its expression.

To transiently activate *kRASG12V* expression (Fig. 1E), we injected a *ubi:Eos; UAS:kRASG12V-T2A-CFP* plasmid together with *Tol2* transposase mRNA into *Tg(ubi:Gal4-ERT2)* embryos at the one-cell stage. Introducing cyclofen or photo-activating caged-cyclofen releases the Gal4-ERT2 protein from its chaperone complex and turns on the expression of *kRASG12V*. Once cyclofen is removed (or diffuses out following photo-activation) the expression of the oncogene is turned off and its products (mRNAs and proteins) are slowly degraded, resulting in a transient expression of *kRASG12V* (Fig. S1A,C,E).

To constitutively activate the oncogene, we designed a photo-activable Cre/loxP recombination system (Fig. 1F). Either *UAS:Cre* and *ubi:loxP-Eos-stop-loxP-kRASG12V-T2A-mTFP* plasmids were co-injected into *Tg(ubi:Gal4-ERT2)* embryos, or *ubi:loxP-Eos-stop-loxP-kRASG12V-T2A-mTFP* plasmid was injected into *Tg(ubi:Cre-ERT2)* embryos at the one-cell stage. Adding (or photo-activating) cyclofen activates the recombinase that remodels the genome so that expression of *kRASG12V* is turned on constitutively (Fig. S1B,D,F).

**Figure 2:**
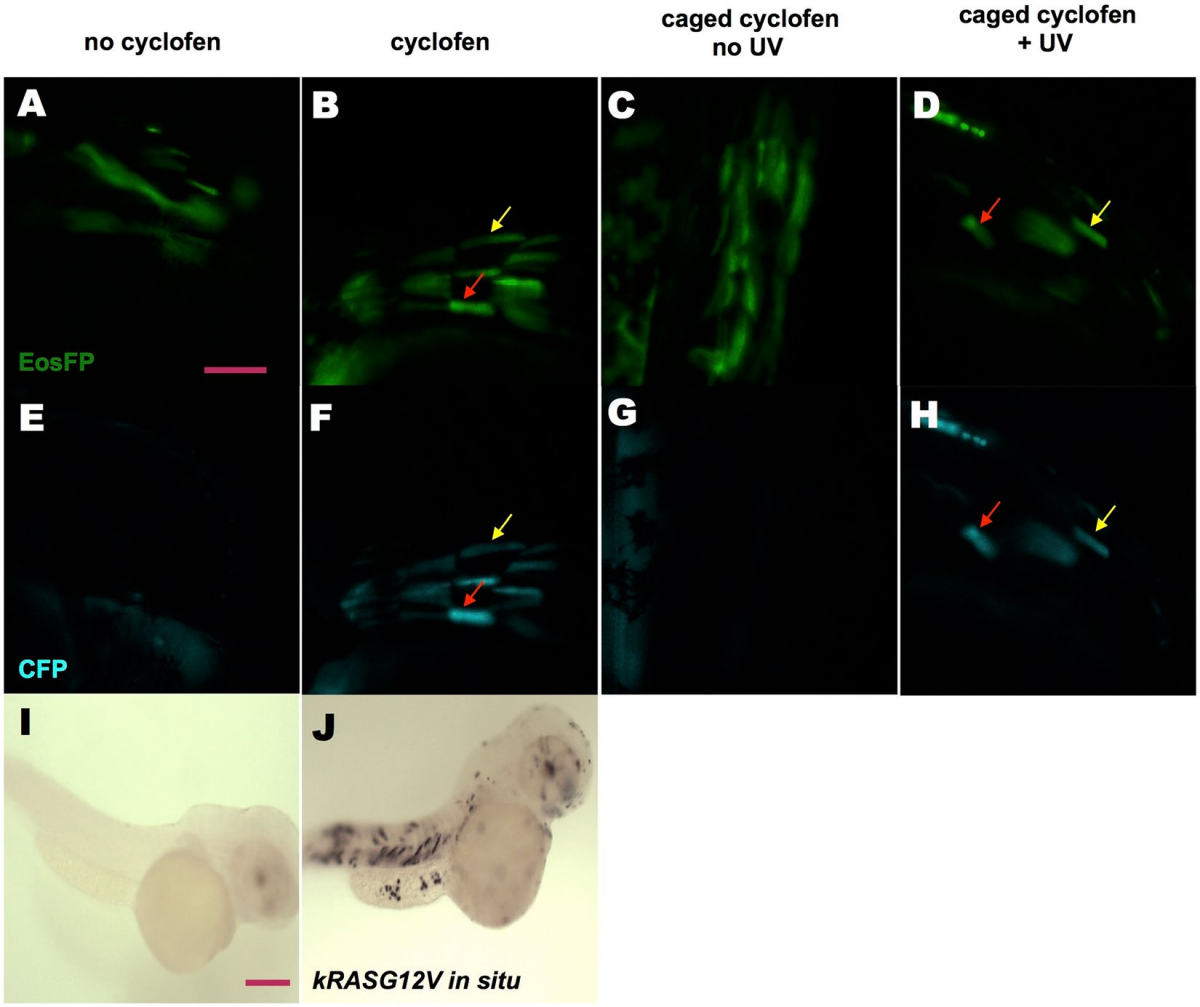
*KRASG12V* induction by incubation in cyclofen or photo-activation of caged cyclofen. (**A**,**E**) Injecting *UAS:kRASG12V-T2A-CFP; ubi:Eos* plasmid into *Tg(ubi:Gal4-ERT2)* embryos resulted in a mosaic expression of EosFP protein, but (in absence of free cyclofen) no expression of CFP. (**B**,**F**) Embryos treated with 2 μM cyclofen at 32hpf display expression of CFP co-localized with EosFP expression (denoted by arrows). (**C**,**G**) Incubation of caged cyclofen (with no UV) did not activate CFP expression while (**D**,**H**) 2 min UV uncaging effectively activated the expression of CFP 12 hours later (denoted by arrows). The induction of *kRASG12V* by cyclofen was confirmed by *kRASG12V* mRNA *in situ* hybridization. Human *kRASG12V* mRNA was transcribed only in the embryo incubated in cyclofen (**J**), but not in the control embryo (**I**). Scale bar: 200 μm.

First, we wanted to confirm that the approach described above allowed for (photo-) activable expression of *kRASG12V*. To test this, we injected a *ubi:Eos; UAS:kRASG12V-T2A-CFP* plasmid into *Tg(ubi:Gal4-ERT2)* embryos at the one-cell stage. Healthy, normal embryos were selected at 24 hpf (hours post fertilization) and were transferred to fresh E3 medium. At 32 hpf, some injected embryos were incubated with 2 μM cyclofen, while others were left in the E3 medium as control. Twelve hours later, despite auto-fluorescence from the yolk, substantial CFP expression was only detected in the cells of embryos treated with cyclofen (Fig. 2B,F), but not in the control embryos (Fig. 2A,E). The fluorescence of CFP co-localized with the fluorescence of EosFP (Eos fluorescent protein), indicating that, as designed, they were co-expressed in the same cell (denoted by arrows). Beyond fluorescent markers, we also looked for more direct evidence of kRASG12V expression. To that purpose we used *in-situ* hybridization to probe for the presence of kRASG12V mRNA in embryos activated or not with cyclofen. Indeed, the oncogene mRNA could only be detected in the injected embryos treated with cyclofen (Fig. 2J), but not in the embryos without cyclofen treatment (Fig. 2I). After verifying that the expression of the oncogene could be induced by cyclofen, we tested the photo-control of transient oncogenic expression. The same injected embryos were incubated at 32 hpf in 4 μM caged cyclofen for 4 hours. They were then rinsed and transferred into fresh E3 medium. To photo-activate the caged cyclofen, the embryos were illuminated for 2 minutes with a benchtop UV lamp (~365 nm). Twelve hours later, injected embryos treated with caged cyclofen without UV illumination expressed only EosFP, but no CFP (Fig. 2C,G). Embryos that were UV illuminated displayed a clear expression of CFP that co-localized with EosFP expression (Fig. 2D,H), demonstrating the successful photo-induction of kRASG12V expression. We also validated both the transient and constitutive expression systems by analyzing the expression over-time of *kRASG12V* and fluorescent markers (Supplementary Information and Figs S1, S2).

### Tumor induction by transient and constitutive expression of kRASG12V

**Figure 3:**
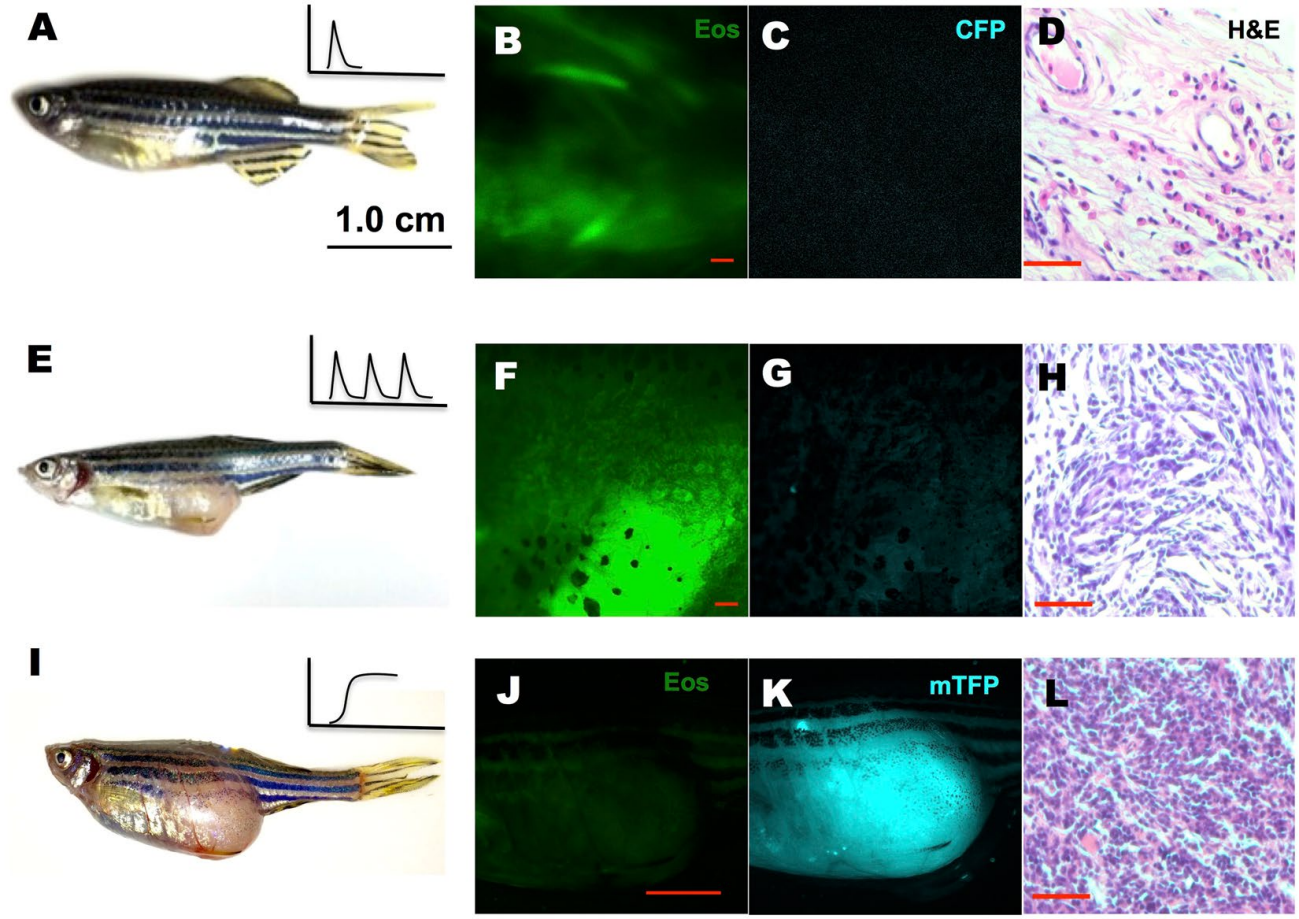
Tumor induced by periodic or constitutive, but not transient activation of *kRASG12V* expression. In the latter system, one-time activation of *kRASG12V* expression was induced by transient cyclofen incubation or photo-activation of caged cyclofen at 1dpf (**A**). Periodic *kRASG12V* expression was achieved by 1 day incubation in 2 μM cyclofen every 5 days for a course of two months (**E**). (**B**–**D**) Transient induction of *kRASG12V* did not affect normal development (as shown by the histopathological staining) with no CFP expression in one-year old fish. (**E**–**H**) Tumors were observed (albeit rarely) in fish periodically treated with cyclofen. A representative one-year old fish displayed a clear tumor (**E**) revealed by histopathological staining (**H**). Interestingly, the cells in the tumor still expressed EosFP (**F**), but lost CFP expression (**G**). (**I**–**L**) Constitutive *kRASG12V* expression following light-activation of caged cyclofen induced tumor (**I**) in 5 month-old fish as revealed by both fluorescent markers (**J**,**K**) and H&E staining (**L**). Scale bar: B, D, F, H, L 200 μm; J, 1 cm.

Having validated our photo-activation approaches, we tested the possibility of inducing a tumor by expressing the oncogene in zebrafish. First, a transient activation of *kRASG12V* was performed in 1 dpf *Tg(ubi:Gal4-ERT2)* embryos injected with a *ubi:Eos; UAS:kRASG12V-T2A-CFP* plasmid at the one-cell stage and incubated for 4 hours in cyclofen or caged cyclofen with photo-activation. Since injection procedures sometimes result in developmental defects, we only selected healthily developing embryos at 3 dpf for further observations. We observed no obvious tumorigenesis up to 10 dpf. Fish were then housed in regular fish system and monitored biweekly for gross tumor morphology. Even after one year, no tumors were detected in any of the 82 cyclofen treated fish or the 68 photo-induced fish that we monitored. Representative fish were taken out for imaging and pathological analysis. We found normally developing fish (Fig. 3A) with visible expression of EosFP (Fig. 3B), but no more CFP (Fig. 3C). Hematoxylin-Eosin (H&E) staining also showed no noticeable tumor histology (Fig. 3D). Since a pulse of *kRASG12V* induction at 1 dpf failed to induce a tumor, we attempted to activate *kRASG12V* expression at an earlier stage. Thus 2 μM cyclofen was added immediately after injection at the one-cell stage for 24 hours. Again, only healthy embryos were selected at 3 dpf, and embryos were carefully monitored for 2 weeks. We found severe developmental defects among many (23/124) cyclofen treated fish through 5–8 dpf (Fig. S3A & B). In addition, a small portion (9/124) of the fish displayed obvious tumorigenic phenotypes, with hyperplasic tissues strongly expressing CFP (and thus the oncogene) even at 6 dpf (Fig. S3C–E). All the embryos that displayed severe developmental or tumorigenic defects did not survive their first two weeks. However, all the fish that survived grew normally with no apparent tumors detected over a year. These results imply that a short pulse of *kRASG12V* expression before 1 dpf is not able to induce a tumor as the fish grows into adulthood. We then wondered whether prolonged activation of *kRASG12V* expression could make a difference. Thus we periodically activated *kRASG12V* by treating the fish in 2 μM cyclofen for 24 hours every five days for two months. By the end of these two months, while the fish did display CFP fluorescence (and thus co-expression of *kRASG12V*), we did not observe any evident tumors. The fish were subsequently left growing under normal aquatic condition. Within a period of 12 months, a few of these fish (2/64) did develop tumors. A representative tumor (Fig. 3E) near the urinary bladder was excised for H&E staining (Fig. 3H). We observed a pack of homogeneous cells with condensed nuclei and increased nuclei-cytoplasm ratio. These cells are disorganized and do not display the texture of normal tissue in the same region (Fig. 3D). These histological characteristics reproduce the typical morphology and growth patterns of tumors seen in zebrafish^26^. Notice however that although the histo-pathological examination revealed typical tumor morphology, the expression of CFP (and the concomitant oncogene) was no longer detected (Fig. 3F,G).

In parallel to transient activation of *kRASG12V*, we also investigated tumor development caused by constitutive expression of the oncogene. To induce its constitutive activation, *ubi:loxP-Eos-stop-loxP-kRASG12V-T2A-mTFP* plasmid together with *Tol2* mRNA were injected into *Tg(ubi:Cre-ERT2)* embryos at the one-cell stage. Healthy embryos at 1 dpf expressing EosFP were treated with 2 μM cyclofen or with 4 μM caged cyclofen for 4 hours. Those embryos were then transferred to E3 medium and photo-activation was conducted with 2-minute illumination by a UV lamp. Tumorigenic phenotypes in some embryos could be readily observed at 5 dpf (Fig. S4). Moreover, tumors were observed in adult fish (8/76) through 2 months to 1 year. High expression of mTFP was also detected in those tumors (Fig. 3I–L and Fig. S5).

### Characterizing the correlation between the probability of tumorigenesis and the number of *kRASG12V* expressing cells

**Figure 4:**
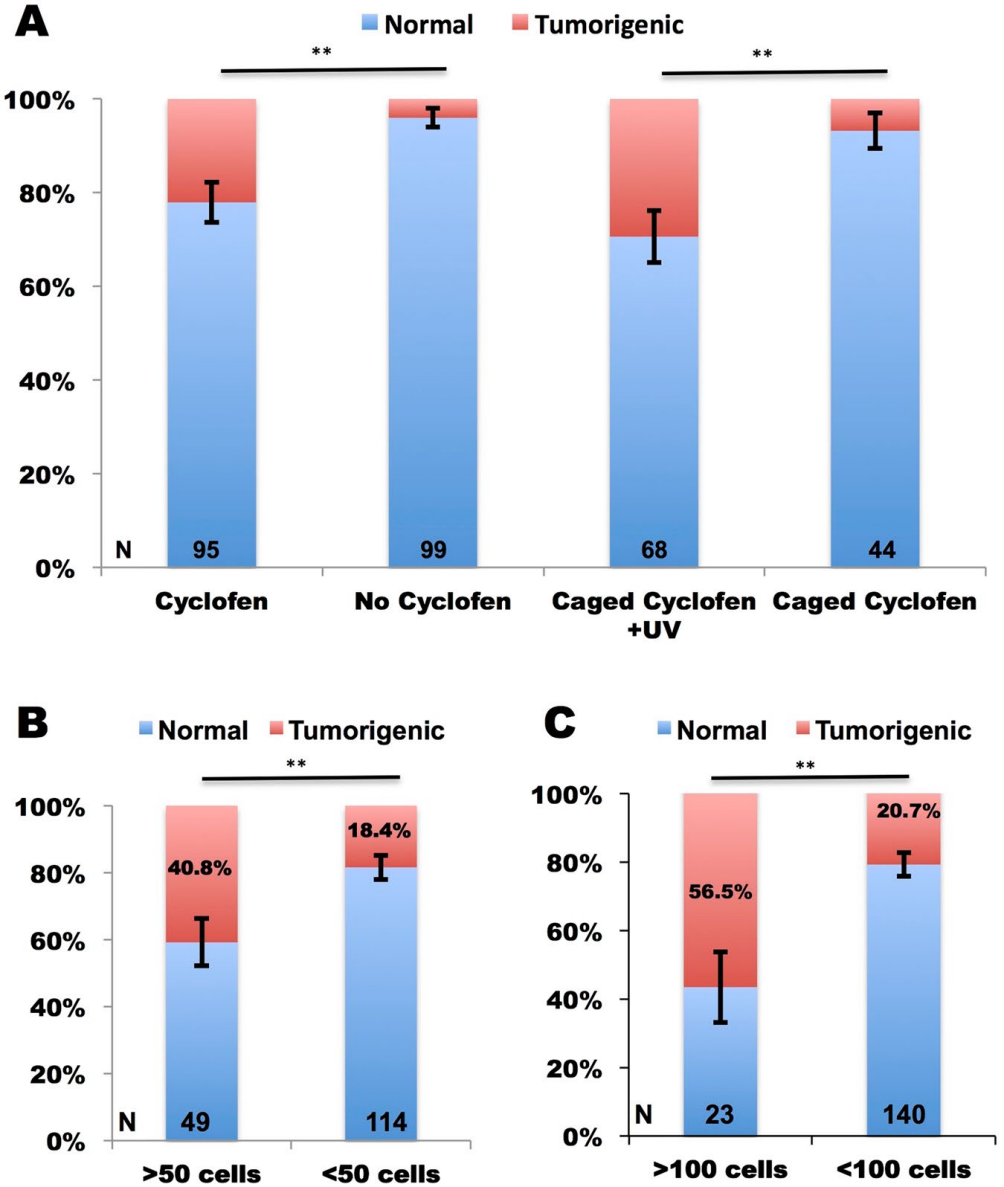
Statistics of early tumorigenesis in embryos with constitutive activation of *kRasG12V*. *Tg(ubi:Cre-ERT2)* embryos injected with *ubi:loxP-Eos-stop-loxP-kRASG12V-T2A-mTFP* plasmid were split into cyclofen, no cyclofen, caged cyclofen + UV and caged cyclofen groups. Tumorigenic probabilities were measured for each group from 3 dpf to 10 dpf (**A**). For the cyclofen group, embryos were separated into four sub-groups based on the number of cells expressing mTFP at 3dpf (more or less than 50 (**B**) and more or less than 100 cells (**C**)), and tumorigenic probabilities were assessed for each sub-group (**B,C**). Statistical significance: **p < 0.01.

Thus far, we have seen that constitutive induction of *kRASG12V* expression in early developing embryos, sometimes resulted in tumor growth. We wondered why certain fish, but not others developed a tumor, even while the oncogene was turned on constitutively in all the embryos. Our initial hypothesis was to link tumorigenic probability to the number of cells in which *kRASG12V* expression was activated. To test the hypothesis, we only focused on constitutive activation of *kRASG12V* since transient activation was much less efficient in causing tumorigenesis. In this experiment, we injected *ubi:loxP-Eos-stop-loxP-kRASG12V-T2A-mTFP* plasmid together with the *tol2* mRNA into *Tg(ubi:Cre-ERT2)* embryos at the one-cell stage. This resulted in mosaic embryos expressing the Eos protein in some of their cells. Constitutive expression of *kRASG12V* (and the concomitant expression of mTFP) was activated at 1 dpf by either cyclofen or photo-activation of caged cyclofen. Only healthily developing embryos at 3 dpf were selected for continuing analysis, and tumorigenesis probability was measured till 10 dpf. Here, tumorigenesis was defined when obvious hyperplasic tissues were observed. First we noticed that activation of *kRASG12V*, either by native cyclofen or by photo-activation of caged cyclofen, resulted in significant increase of tumorigenesis (Fig. 4A). Then following cyclofen activation we split the embryos into four subgroups based on the number of mTFP-expressing cells they had at 3 dpf: two sub-groups with more (or less) than 50 cells and two sub-groups with more (or less) than 100 cells. We found that the groups with more mTFP-expressing cells at 3 dpf exhibited a much higher tumorigenic rate than the groups with less mTFP-expressing cells (Fig. 4B,C). Besides the observation of a high correlation between the number of cells expressing *kRASG12V* and early tumorigenesis probability, we used these data to estimate the probability of tumorigenesis for a single transformed cell, assuming every cell could equally contribute to tumorigenesis. Thus for the 50-cells groups, the upper bound on the probability can be estimated as (see Fig. 4B): ${{\rm{P}}}_{50}^{{\max }}$ = 40.8/50 × 100% = 0.82%, and the lower bound as ${{\rm{P}}}_{50}^{min}$ = 18.4/50 × 100% = 0.37%. Similarly, based on the 100-cell groups, we estimated the upper and lower bound probability as 0.56% and 0.21%. Thus, we deduce that the probability of tumorigenesis from a single cell is about 0.5%.

### Precise optical activation of *kRASG12V* expression in live zebrafish embryos

**Figure 5:**
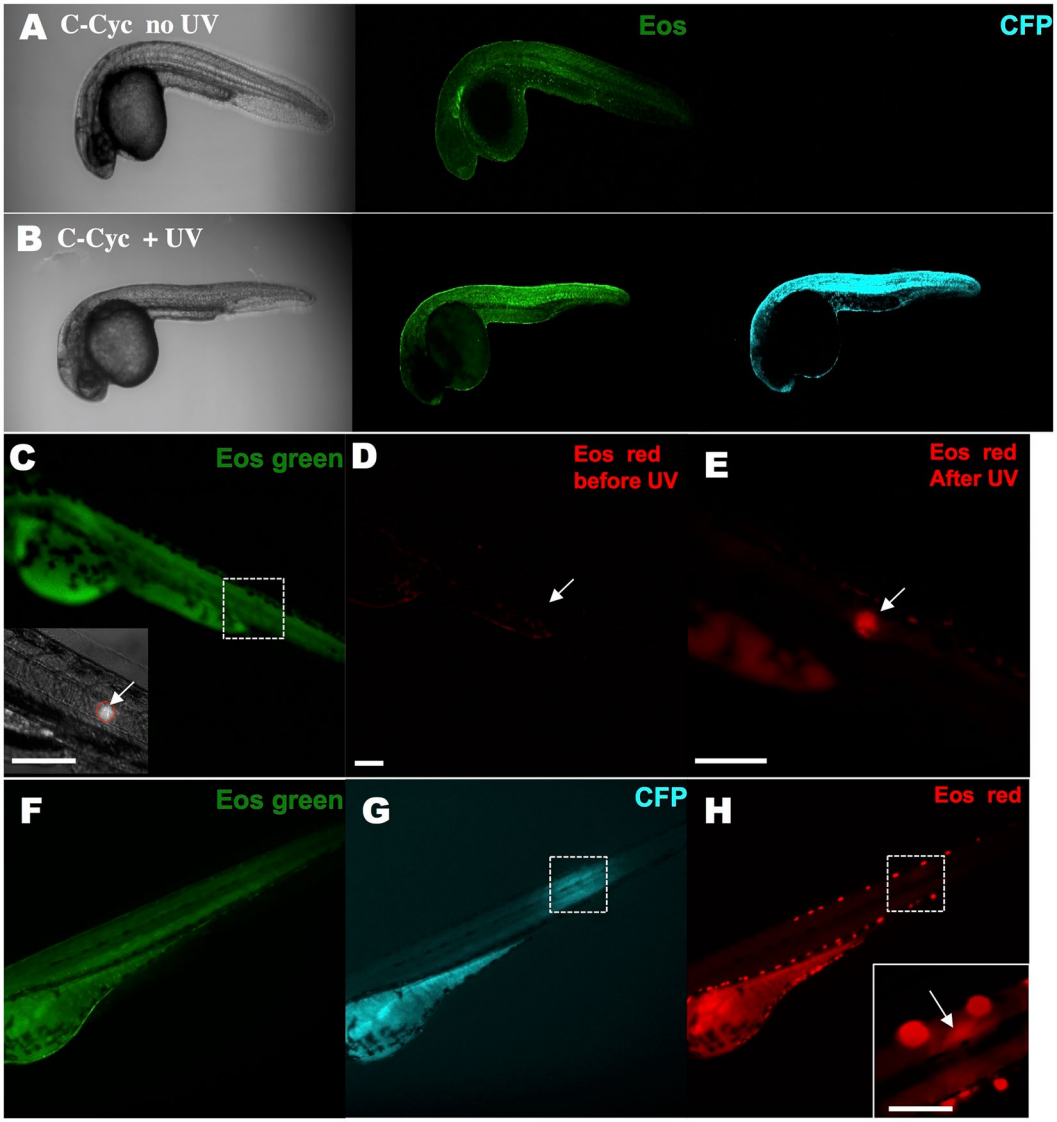
*KRASG12V* activation and tracking in a small group of cells within a live zebrafish embryo. A stable *Tg(ubi:Eos;UAS:kRASG12V-T2A-CFP)* line was generated and crossed with the *Tg(ubi:Gal4-ERT2)* line. Caged cyclofen alone at 1 dpf was not able to induce CFP expression (**A**), while 2-minute UV illumination (**B**) effectively turned on CFP after 18 hours. (**C**) Local activation of *kRASG12V* expression was achieved by introducing a focalized UV beam onto embryos pre-incubated with caged cyclofen. (**D**,**E**) 1 minute UV effectively converted EosFP from green to red. (**F**,**G**) After 18 hours, CFP expressed in a localized region of the illuminated embryo and weak remaining EosFP red signal was detected in the same region (**H**). Scale bar: 200 μm.

The fundamental challenge in understanding cancer initiation and evolution lies in the difficulties identifying and visualizing the very early tumorigenic event in single cells. We therefore investigated whether our method could precisely enable spatio-temporal targeting of *kRASG12V* activation. In this case, a stable *Tg(ubi:Eos;UAS:kRASG12V-T2A-CFP)* fish line was generated and crossed with the *Tg(ubi:Gal4-ERT2)* line. Again, we showed that caged cyclofen only was not able to activate CFP expression at 1 dpf (Fig. 5A), but that 2 minutes global UV illumination could effectively turned on CFP expression (Fig. 5B). Then we tested local light activation of transient *kRASG12V* expression by photoactivation of caged cyclofen. We generated a focalized beam of UV light (a circular region about 40 μm in diameter; peak around 396 nm) within the 1dpf embryo (Fig. 5C). The photo-switchable EosFP protein^27^ was efficiently converted from green to red after 1 min UV illumination (Fig. 5D,E indicated by arrows). Eighteen hours later, we observed local expression of CFP in the illuminated region implying local activation of *kRASG12V* expression (Fig. 5F,G). While the expression of the photo-activated EosFP red decreased over time, it remained correlated with the expression of CFP (Fig. 5H), indicating a successful photo-activation of *kRASG12V* expression in the illuminated region only. Furthermore, we also tested the activation of *kRASG12V* expression at different developmental stages by a UV laser illumination. We illuminated a small square region (40 × 40 μm) for 5-second with the 405-nm laser line. Embryos were thus photo-activated at 10 hpf (Fig. S6A) or 32 hpf (Fig. S6B,C) within different tissues. Clear local expression of CFP indicated precise light activation of oncogene expression. In agreement with our previous experiments with transient expression of *kRASG12V*, we found that local transient activation of the oncogene led to normal development, and all embryos developed into adults without any tumorigenic phenotypes. In contrast, global photo-activation of *kRASG12V* expression at 10 hpf resulted in developmental defects at 5dpf (Fig. S6D) in many embryos (24/87), and hence their death within 10 days.

**Figure 6:**
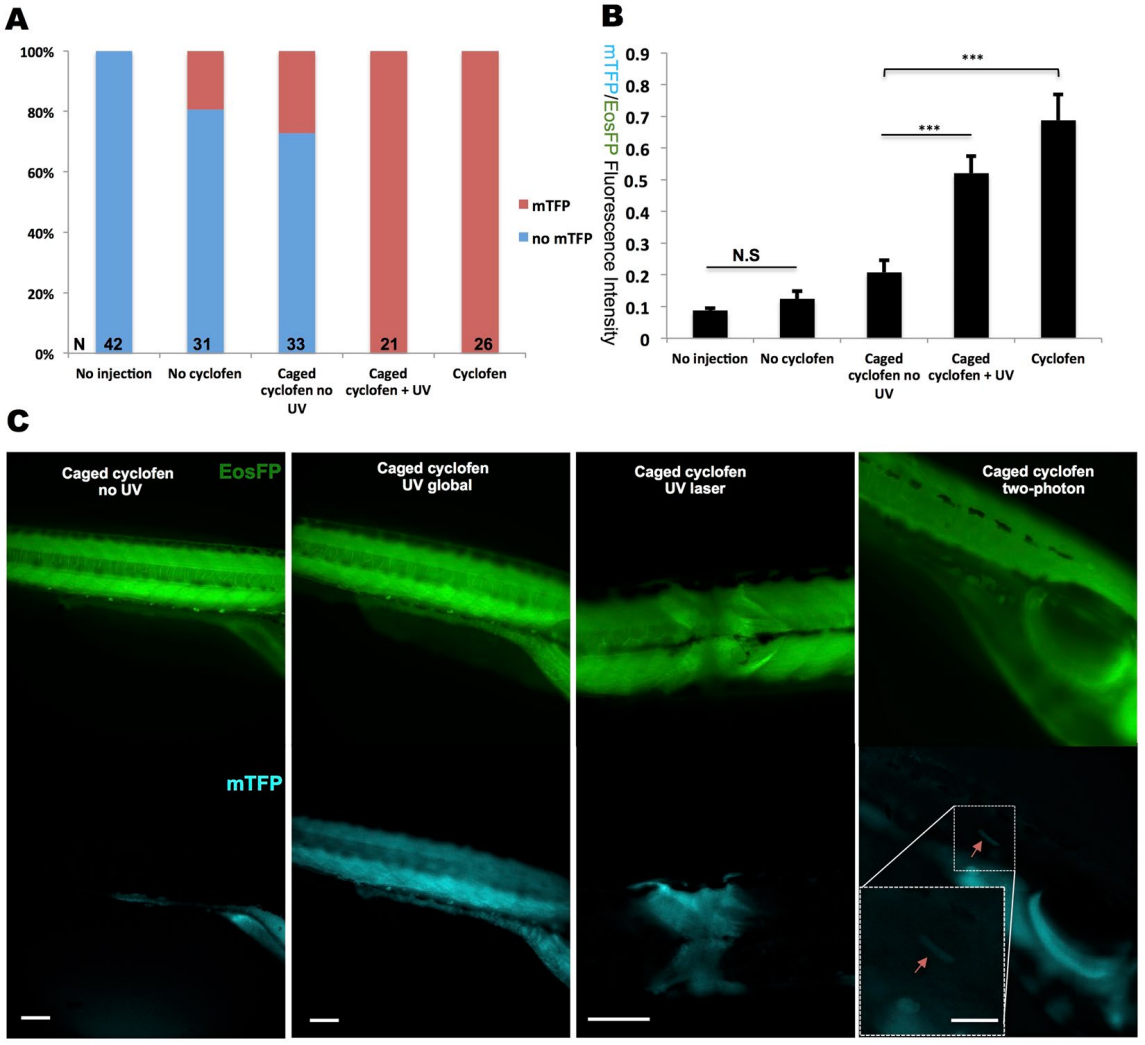
Constitutive photo-activation of *kRASG12V* expression in localized tissue and single cells. To induce constitutive activation of *kRASG12V*, *Cre-ERT2* mRNA was injected into *Tg(ubi:loxP-Eos-stop-loxP-kRASG12V-T2A-mTFP)* embryos at the one-cell stage. (**A**) Despite some minor light independent activation of Cre, mTFP expression could be fully induced by both cyclofen and caged cyclofen photo-activation. (**B**) Quantification of *kRASG12V* activation signal via the ratio of the fluorescence of the co-expressed mTFP protein and the (partially) floxed Eos protein. (C) UV (lamp or laser) and two-photon activation were performed at 10hpf to achieve global (with the UV lamp), local (with the UV laser) and single cell (with 2-photon laser) activation. Fish were imaged at 2dpf. Scale bar: 200 μm. Data are presented as mean ± SEM. ***p < 0.001; N.S, not significant.

To allow precise constitutive activation of *kRASG12V* expression by light, we also generated a stable *Tg(ubi:loxP-Eos-stop-loxP-kRASG12V-T2A-mTFP)* line. We first crossed this line with the *Tg(ubi:Cre-ERT2)* line, but found that mTFP expression could be hardly detected after cyclofen activation (Fig. S7A). Since we also observed a low EosFP expression, we suspected that crossing these two lines-resulting in a double heterozygous line- would decrease the expression of both Cre-ERT2 and fluorescent proteins. To circumvent this issue, we directly injected about 20 pg *Cre-ERT2* mRNA into the *Tg(ubi:loxP-Eos-stop-loxP-kRASG12V-T2A-mTFP)* embryos at the one-cell stage. Although we found about 20–30% of injected embryos showed leakage of mTFP expression without photo-activation (probably due to too large a dose of injected *Cre-ERT2* mRNA), most of the injected embryos expressed mTFP only after cyclofen activation or caged cyclofen photo-activation (Fig. 6A). Distinct fluorescent patterns of non-activated, leaky and globally-activated embryos are shown in Fig. S7B. We also measured the mTFP/Eos fluorescent ratio to quantify the signals. The non-injected group set the auto-fluorescence baseline. Despite minor leakage, the mTFP/Eos ratio could be significantly increased by both cyclofen and photo-activation of caged cyclofen (Fig. 6B). Local activation was performed with 405 nm laser at 10 hpf using the Leica SP5-Blue microscope as before. We found that a 5-second laser illumination locally activated mTFP expression with a notable decrease of EosFP fluorescence in the same region, confirming recombination by Cre and activation of the oncogene (Fig. 6C).

To achieve local (possibly single cell) control of gene expression with this approach, a possible concern is the diffusion of the cyclofen molecule upon photo-activation for a prolonged period. Indeed the diffusion constant of cyclofen is about 400 μm^2^/sec.^28^. Illumination with a weak UV source for 5 sec or 1 min (as mentioned earlier) results in cyclofen uncaging over a region of typical radius ~50 μm or ~160 μm respectively. Such diffusion can make the light activation of *kRASG12V* expression in a single cell challenging. One solution is the use of a stronger localized UV illumination (e.g. a focused UV laser beam) over a shorter time (<1 sec). Another possibility is to use two-photon uncaging, which has been shown to result in the release of cyclofen in a much more confined region, down to a single cell^25^. To test that later option, we performed two-photon activation to reach single cell resolution. We injected *Cre-ERT2* mRNA into *Tg(ubi:loxP-Eos-stop-loxP-kRASG12V-T2A-mTFP)* embryos at the one-cell stage. The embryos were subsequently photo-activated at 10 hpf for 10 seconds with the two-photon (750 nm) beam of the microscope. At 2 dpf, weak mTFP expression in single cells could be detected (Fig. 6C noted by arrow). For the 19 embryos tested for two-photon activation, we found that 6 displayed single cell mTFP expression, 3 showed leaky expression (i.e. mTFP expression in non-activated cells) and 10 had no any mTFP expression (Fig. S7C). While two-photon activation led to higher mTFP/Eos fluorescence ratio than for the non-activated group, the mTFP/EOS ratio was still significantly lower than by direct cyclofen activation (Fig. S7D).

To test whether global and local activation would induce tumor development, those activated fish that developed normally were kept growing to adulthood. However, although mTFP was still expressed in those fish, no tumor was observed within one year. We found that the expression of *kRASG12V* in these fish was significantly lower at 5dpf and 5mpf than in those that were injected with the equivalent plasmid (Fig. S8A), as estimated from both the mTFP raw intensity (Fig. S8B) and the mTFP/Eos fluorescence ratio (Fig. S8C).

## Discussion

A variety of approaches to control protein activity by light have been developed for over a decade^29^. These methods have greatly accelerated our understanding of biological systems. However, as far as *in vivo* applications in live organisms are concerned, these tools have been used mainly in neuroscience^30^. Most optogenetic approaches adopt light-sensitive proteins that usually have some activity even without light^29^ and may require long (hours) illumination times^31–33^. This critical drawback makes it rather difficult to precisely photo-manipulate biological processes such as cancer induction. In this work, we demonstrate a powerful new optical tool to manipulate tumor induction in live zebrafish. Our approach utilizes caged-cyclofen, a synthetically modified estrogen receptor inducer, which allows for stringent light-dependent activation of proteins fused to the binding domain of the modified estrogen receptor (ERT2). In absence of ligand these protein constructs are sequestered by cytoplasmic chaperones. Uncaging of the ligand (cyclofen) releases the fused proteins from their chaperone complex, allowing them to diffuse into the nucleus and control the expression of the desired genes.

Our experiments highlight two inducible systems for the transient or constitutive expression of *kRASG12V*. We validated these two systems by micro-injection of the appropriate gene construct at the one-cell stage, obtaining mosaic embryos expressing the oncogene in some of their cells. We find that a short pulse (less than 24 hours) of *kRASG12V* expression at 1 dpf in those embryos fails to induce tumor formation. This is not so surprising since cancer results from a prolonged process of cumulative mutations^34, 35^. The decrease of CFP fluorescence within a few days confirms the loss of *kRASG12V* expression and activity. Therefore a time-limited activation of *kRASG12V* might not be enough to drive further oncogenic mutations, hence no tumor. Notice however that an early (<1dpf) short pulse of *kRASG12V* results in severe developmental defects and embryonic lethality. These phenotypes are probably the result of the teratogenic effects of over-expression of *kRASG12V*, which is known to play a critical role during early embryogenesis. Notice that embryonic defects caused by *kRASG12V* over-expression have been previously reported in both mouse^36^ and zebrafish^37^.

In contrast, constitutive activation of *kRASG12V* at >1dpf, does not result in noticeable developmental defects but increases early tumorigenic rates and causes tumor in adult fish. Surprisingly, we find that periodic activation of *kRASG12V* for 2 months (24 hours every 5 days) does not lead to tumor formation by the end of the treatment. However, a few fish (2/64) do develop tumors (albeit without CFP and hence kRas expression) after one year. This result suggests that periodic, but not necessarily constitutive activation of the oncogene could also result in tumorigenesis. The precise mechanism that underlies this phenomenon needs further elucidation.

On the other hand, permanent activation of *kRASG12V* from 1dpf in mosaic embryos causes early tumorigenesis with a probability that correlates with the number of cells expressing the oncogene. This is expected because the more numerous the cells with oncogenic activation the greater the probability of passenger mutations and tumor growth. This result is also in agreement with previous finding that reported significant liver hyperplasia and tumor after continuous induction of *kRASG12V* in the zebrafish liver^11^. By examining the number of cells expressing mTFP at 3dpf and analyzing the probability of tumorigenesis at 10dpf, we estimated that the probability of tumorigenesis due to a single transformed cell is as low as 0.5%. The analysis assumes that in this specific case every transformed cell can equally contribute to early tumorigenesis. However since the transgenic line did not develop tumors following activation of the oncogene, we deduce that the aforementioned probability may depend on the strength of oncogene expression, context, cell type, etc. This important issue deserves further study.

In the transgenic lines we engineered, our approach has shown precise control of *kRASG12V* induction in different regions and single cells of developing zebrafish embryos. This level of control has not been achieved by any other inducible cancer models reported so far. However, we find that the constitutive photo-activation of *kRASG12V* in this transgenic line is not efficient to induce a tumor. The lower expression level of mTFP in the photo-activated transgenic fish as compared to the injected fish (with mosaic expression of the oncogene) points to a lower expression of the oncogene. This fact may explain why these fish, even with active oncogenic expression, fail to develop tumors within one year. Our observations suggest that not only the number of oncogene expressing cells, but also the level of expression of the oncogene may be important in contributing to tumor induction. Engineering a similar line albeit with a stronger promotor should allow us to investigate that important issue.

In summary, our study presents the first light-inducible tumor model in a vertebrate. We have demonstrated the success of light activation of the human *kRASG12V* oncogene in the zebrafish and have observed the multiple tumors formed over a one-year period. Our technique should be helpful in evaluating how different organs and tissues respond to the same oncogenic induction. More importantly, successful demonstration of activating and labeling *kRASG12V* expression in a small number of cells by light makes this technique powerful in the study of the early initiation and evolution of cancer. This novel light-inducible model should help investigate and understand cancer initiation, and ultimately lead to the development of novel and effective barriers to, and treatments for cancer.

## Materials and Methods

### Plasmid construction and *in vitro* transcription

A 564 bp DNA sequence of human *kRASG12V* was slightly modified to optimize its expression in zebrafish (see below for a full sequence). *Cre*, *kRASG12V* and *loxP-Eos-stop-loxP-kRASG12V-T2A-mTFP* sequences were ordered from Eurogentec and were inserted in pUC57 vector. The *ubi:Eos; UAS:kRASG12V-T2A-CFP* plasmid was cloned by inserting the *kRASG12V* sequence into a *UAS:fgf8a-T2A-CFP; ubi:Eos* plasmid (unpublished; construct sequence is available upon request), using the KpnI and FspAI restriction sites to replace the fgf8a. Similarly, we made the *UAS:Cre;ubi:Eos* plasmid by replacing *fgf8a-T2A-CFP* with *Cre*. Based on the *UAS:fgf8a-T2A-CFP; ubi:Eos* plasmid, *ubi:loxP-Eos-stop-loxP-kRASG12V-T2A-mTFP* plasmid was cloned by first replacing *Eos* with *loxP-Eos-stop-loxP-kRASG12V-T2A-mTFP* sequence, using BamHI and EcoRV sites, and then by deleting the *fgf8a-T2A-CFP* sequence, using KpnI and FspA1 sites, followed by quick blunting and ligation. A *ubi:Gal4-ERT2* plasmid was derived from the *ubi:Cre-ERT2* plasmid^24^ by substituting Cre sequence with Gal4-FF between NcoI and SmaI. All nucleotide sequences and details of the cloning steps are available on request. To make *Tol2* transposase mRNA, a pCS-TP *Tol2* plasmid was first linearized with NotI and was used to synthesize mRNA using a mMESSAGE mMACHINE^®^ SP6 Transcription Kit (Thermo Fisher Scientific). Similar to *Tol2* mRNA, *Cre-ERT2* mRNA was made with another pCS*Cre-ERT2* plasmid.

### Fish lines and maintenance

With the plasmids we constructed, we generated three stable fish lines via the *Tol2* transposon system^38^: *Tg(ubi:Gal4-ERT2), Tg(ubi:Eos;UAS:kRASG12V-T2A-CFP)* and *Tg(ubi:loxP-Eos-stop-loxP-kRASG12V-T2A-mTFP)*. The transgenic line *Tg(ubi:Cre-ERT2)* ^39^ was kindly provided by Dr. D. Traver. All the fish lines were raised at UCLA Zebrafish Core Facility. The facility also provided regular maintenance of all zebrafish lines we generated. The overall health of the animals in the fish-facility is under the supervision of UCLA veterinary advice. Animal care and all experimental procedures were performed in accordance with the protocol approved by UCLA Institutional Animal Care and Use Committee (ARC# 2001–074–33).

### Drug treatments and UV uncaging

The Jullien lab synthesized native cyclofen and caged cyclofen in powder form. For long-term storage, both native and caged cyclofen were kept at −80 °C. Stock of 2 mM cyclofen and 4 mM caged cyclofen in DMSO were prepared and also kept at −80 °C in darkness. To induce *kRASG12V* expression, embryos were either directly incubated in 1, 2, 10 μM native cyclofen for 2–24 hours, or incubated in 4, 10 μM caged cyclofen for 2–24 hours and photo-activated by UV before being transferred back to E3 medium. Periodic induction of *kRASG12V* expression was carried out by treating *Tg(ubi:Gal4-ERT2)* embryos injected with *UAS:kRASG12V-T2A-CFP;ubi:Eos* plasmid with 2 μm cyclofen for 24 hours every five days until two-month post fertilization. For whole embryo uncaging, we used a benchtop UV lamp (Fisher VL-6-L) which emits a peak wavelength at 365 nm with a FWHM (full width at half maximum) of 40 nm, and delivers on the illuminated sample a typical photon flux of ~4.3 Einstein/(s·m^2^)^40^. Injected embryos pre-treated with caged cyclofen were briefly rinsed in E3 medium before they were illuminated by the benchtop UV lamp for 2 minutes in a 60 mm × 15 mm petri dish. Illuminated fish were transferred back to E3 medium, covered with aluminum foil and were incubated at 28 °C. Localized uncaging was achieved on a Nikon microscope equipped with an adjustable iris and a lumencor LED light source with a UV light peaking at 396 nm. The maximum relative spectral power of the UV light was 27 mW/nm. The size of the uncaging region was controlled by an iris that defines a circular illumination area ranging from 50 μm to 400 μm in diameter. Uncaging was performed for 20 seconds to 2 minutes at the maximum power of the UV LED light on each embryo. After uncaging, embryos were transferred to a 12-well plate with E3 medium and incubated in total darkness. Laser uncaging was conducted with the 405 nm laser equipped on a Leica SP5-BLUE confocal microscope (UCLA CNSI core facility). Two-photon activation was performed using a Ti-Sapphire laser^25^ with a 40×, 0.8 NA water immersion objective. The laser beam (200 fs, 76 MHz, 20 mW, 750 nm) was used to photo-activate caged-cyclofen for 10 s in 10hpf embryos.

### RT-qPCR and *in-situ* hybridization

Expression of *kRASG12V* mRNA was profiled on embryos from one-day to two-weeks post fertilization. At each time point, five to seven injected embryos were collected for mRNA extraction. Total extracted mRNA was purified as previously described^41^, and stored at −80 °C. SuperScript® III First-Strand Synthesis System (Invitrogen) was used to synthesize and purify cDNA from the frozen samples of mRNA. Fast SYBR Green Master Mix (Thermo Fisher Scientific) was used for qPCR. Primer sets used for *Eos* and *kRASG12V* cDNA detection were: Eos forward (GCAACAAAGCCATGTGAATATGA), Eos reverse (CAAACTTTCCCG CCAATGGTCCA), *kRASG12V* forward (AACCAATGTATAGAAGGCATCAT), *kRASG12V* reverse (TAAATGTGATTTGCCTTCAAGAA). To account for priming differences, titrations of the *ubi:Eos; UAS:kRASG12V-T2A-CFP* and *ubi:loxP-Eos-stop-loxP-kRASG12V-T2A-mTFP* plasmids were used as reference templates. The difference (ΔCt) in the number of amplification cycles between *kRASG12V* and *Eos* (used as a reference gene) was measured and normalized to reference templates in triplicate PCR assays. The ratio of the expression of *kRASG12V* to *Eos* was quantified as: 2^−ΔCt^. Whole-mount *in situ* hybridization with digoxigenin-labeled riboprobes was performed at 2 dpf as described previously^42^. Riboprobes for *kRASG12V* mRNA were synthesized from the *UAS:kRASG12V-T2A-CFP* plasmid.

### Microinjection and microscopic imaging

Exogenous genes were incorporated into embryo genomes at the one-cell stage using the *Tol2* transposon system^43^. DNA constructs (final concentration 25 ng/μl) were mixed with *Tol2* mRNA (final concentration 100 ng/μl), and about 200 picoliters mixed solutions were micro-injected into embryos at the one-cell stage, following protocols described earlier^44^. Alternatively, about 200 picoliters *Cre-ERT2* mRNA (final concentration 50 ng/μl) was injected into the *Tg(ubi:loxP-Eos-stop-loxP-kRASG12V-T2A-mTFP)* embryos at the one-cell stage. After injection, embryos were incubated in E3 medium at 28 °C. Healthy, normal embryos were selected at 24 hpf and 3 dpf for further observation and analysis. Fluorescent imaging was performed on a Nikon Eclipse Ti microscope equipped with a C-HGFI Intensilight Fiber illuminator, and confocal imaging was acquired on a Leica SP5-STED microscope. Embryos and adult zebrafish were anesthetized first in tricaine (Sigma-Aldrich), and aligned in a small liquid drop on a cover slide for imaging. The fluorescence intensity of individual images was quantified with ImageJ64 after background subtraction. *In situ* hybridization and H&E samples were imaged on a Leica LMD (Laser Micro Dissection) 7000 Microscope.

### Histological analysis

Ten days post fertilization embryos and adult zebrafish were first anesthetized with tricaine. Selected tissues bearing tumor or normal tissues from control were dissected. The embryos and dissected tissues were fixed in 4% paraformaldehyde overnight at 4 °C prior to rinsing in 70% ethanol. Fixation and decalcification of adult fish were performed as previously reported^45^. Fish samples were then sent to UCLA Translational Pathology Core Laboratory (TPCL) for sectioning and H&E staining. Stained slides were imaged on a Leica LMD (Laser Micro Dissection) 7000 Microscope.

### Statistical analyses

RT-qPCR data were collected from three independent experiments and presented as mean ± standard error of mean (SEM). Statistical significance was analyzed using Fisher’s exact test in Fig. 4, and Student’s two-tailed *t*-test or one-way ANOVA in other figures as appropriate. *P* < 0.05 was indicated as *; *P* < 0.01 was indicated as **; P < 0.001 was indicated as *** and N.S. means “not significant”.

## Electronic supplementary material

Supplementary Information
