# Supplementary material for: Optical Control of Tumor Induction in the Zebrafish
Source: Sci Rep. 2017 Aug 23;7:9195. doi: 10.1038/s41598-017-09697-x (PMC5569104; doi:10.1038/s41598-017-09697-x)
Supplement: Supplementary file 1 — Supplementary Information [file 41598_2017_9697_MOESM1_ESM.pdf]

OPTICAL CONTROL OF TUMOR INDUCTION IN THE ZEBRAFISH

Zhiping Feng,[1,12] Suzy Nam,[2] Fatima Hamouri,[3,4] Isabelle Aujard,[5,6] Bertrand Ducos,[3,4] Sophie Vriz,[7,8] Michel Volovitch,[7,9] Ludovic Jullien,[5,6] Shuo Lin,[10] Shimon Weiss,[1,11] David Bensimon[3,4,11]

[1]Department of Molecular, Cellular and Integrative Physiology, University of California at Los Angeles, Los Angeles, California, USA
[2]Department of Ecology and Evolutionary Biology, University of California at Los Angeles, Los Angeles, California, USA
[3]Laboratoire de Physique Statistique, Ecole Normale Supérieure, PSL Research University, Paris, France
[4]IBENS, CNRS-UMR8197, INSERM-U1024, PSL Research University, Paris France.
[5]École Normale Supérieure, PSL Research University, UPMC Univ Paris 06, CNRS, Département de Chimie, PASTEUR, Paris, France
[6]Sorbonne Universités, UPMC Univ Paris 06, ENS, CNRS, PASTEUR, Paris, France
[7]Center for Interdisciplinary Research in Biology (CIRB), College de France, and CNRS UMR 7241, and INSERM U1050, Paris, France
[8]Department of Life Sciences, Paris-Diderot University, Sorbonne-Paris-Cité, Paris, France
[9]Department of Biology, Ecole Normale Supérieure, PSL Research University, Paris, France
[10]Department of Molecular, Cell and Developmental Biology, University of California at Los Angeles, Los Angeles, California, USA
[11]Department of Chemistry and Biochemistry, University of California at Los Angeles, Los Angeles, California, USA
[12]Present address: Department of Chemical and Systems Biology, Stanford University, Stanford, California, USA
Correspondence: zpfeng@stanford.edu, sweiss@chem.ucla.edu, or david@lps.ens.fr

Supporting Information

Full sequence of the modified human *kRASG12V* is listed below:
atgactgaatataaacttgtggtggttggagctGTTggcgttggcaagagtgccttgacaatccagctgattcagaatcattttgtggacga
atatgatccaacaatcgaggattcctacaggaagcaagtggtgattgatggagaaacctgtctcttggatattctcgacacagcaggtcaaga
ggagtacagtgcaatgagggaccagtacatgaggactggggagggctttctttgtgtgtttgccatcaataatactaaatcatttgaagatattc
accattatagagaacaaattaaaagagttaaggactctgaagatgtgcctatggtcctcgtgggaaataaatgtgatttgccttcaagaacagt
ggacacaaaacaggctcaggacttggcaagaagttatggtattccttttattgaaacatcagcaaagacaagacagggtgttgatgatgcctt
ctatacattggttagagaaatccgaaaacataaagaaaagatgagcaaagatggtaaaaagaagaaaaagaagtcaaagacaaagtgtgt
gattatg

We designed two systems to transiently and constitutively activate *kRASG12V* expression. To prove that these systems worked as designed, we investigated the dynamic patterns of expression in both systems by quantifying the fluorescence of the protein markers as well as the mRNA expression (Fig. S1). For the transient expression approach, *UAS:kRASG12V-T2A-CFP;ubi:Eos* plasmid mixed with *Tol2* mRNA was injected into *Tg(ubi:Gal4-ERT2)* embryos at the one-cell stage. For constitutive activation, we co-injected *UAS:Cre;ubi:Eos* and *ubi:loxP-Eos-stop-loxP-kRASG12V-T2A-mTFP* plasmids into *Tg(ubi:Gal4-ERT2*) embryos together with *Tol2* mRNA at the one-cell stage. In both systems, *kRASG12V* expression was activated at 1 dpf by adding 2 μM cyclofen for 24 hours. We then looked for expression of EosFP, CFP and mTFP over two weeks. Both systems displayed stable EosFP expression over that time. However, for the transient system, CFP expression decreased significantly within less than a week (Fig. S1A). In contrast, mTFP expression stayed fairly stable in the constitutive system (Fig. S1B). Additionally, we quantitatively analyzed the ratio of the fluorescent intensities of the proteins (CFP/EosFP, mTFP/EosFP) over about 50 embryos in each system. The marked decrease of CFP/EosFP ratio in the transient system was obvious after one day and continued to rapidly decrease in the first week (Fig. S1C). On the other hand, cyclofen activation of the Cre/loxP system allowed quite stable mTFP/EosFP ratio over the two-week period studied (Fig. S1D).
Similar measurements on the ratio of mRNA expression (*kRASG12V*/*Eos*) were performed by RT-qPCR on embryos collected at 2 dpf, 3 dpf, 5 dpf and 7 dpf, while 2 dpf embryos without cyclofen activation were used as control. Results on the transient system displayed a gradual decrease over a week of the *kRASG12V*/*Eos* mRNA ratio (Fig. S1E). On the other hand, in the constitutive activation system, the mRNA ratio of *kRAS*/*Eos* was rather constant over that period (Fig. S1F). These results, which agree with the quantification of expression activity by fluorescence, validate the photo-activable approach for both transient and constitutive control of oncogene expression.
We found that co-injection of *UAS:Cre;ubi:Eos* and *ubi:loxP-Eos-loxP-kRASG12V-T2A-mTFP* plasmids into *Tg(ubi:Gal4-ERT2)* embryos displayed some leaky activity of Cre, i.e. mTFP expression without cyclofen activation (Fig. S2A). Nonetheless, the ratio of mTFP/EosFP still significantly increased once the embryos were incubated at 1 dpf for 2 hours in 2 μM cyclofen as compared to the untreated control (Fig. S2B). To achieve constitutive activation of *kRASG12V* with minimal leakage, we injected *ubi:loxP-Eos-stop-loxP-kRASG12V-T2A-mTFP* plasmid into *Tg(ubi:Cre-ERT2)* embryos at the one-cell stage. The results showed that among most embryos (Fig. S2C), incubation in caged cyclofen without UV illumination yielded no detectable mTFP (Fig. S2A). On the other hand, 2 minute global UV uncaging successfully turned on mTFP expression and showed clear disappearance of EosFP expression (Fig. S2A). RT-qPCR analysis

of *kRASG12V*/*Eos* mRNA ratio also showed a significant increase at 2 dpf after cyclofen activation compared with non-irradiated embryos (Fig. S2D). That ratio was stable for a week, indicating effective, yet incomplete recombination. Notice that some embryos expressed global ubiquitous fluorescence of mTFP at 1 dpf (i.e. independent of cyclofen activation), probably due to a high expression of Cre-ERT2 and the incomplete titration of the recombinase by cytoplasmic chaperones. These embryos were discarded and only embryos that did not display any mTFP expression at 1dpf were used for photo-activation experiments (either as control or as response to illumination).

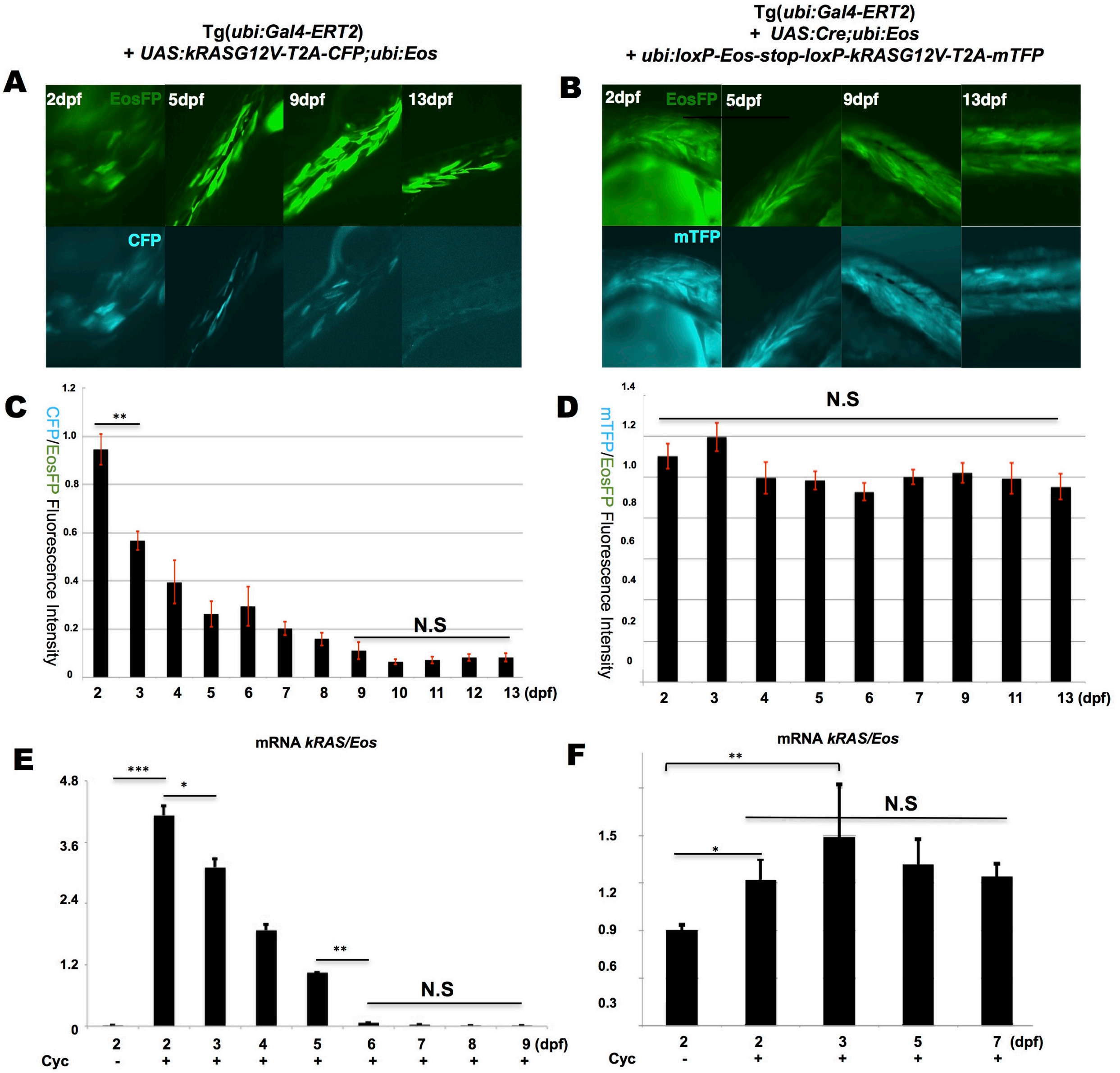

Fig. S1. Dynamic profile of *kRASG12V* expression over time following transient and constitutive cyclofen activation. Transient kRASG12V induction was achieved by injecting *UAS:kRASG12V-T2A-CFP; ubi:Eos* plasmid into *Tg(ubi:Gal4-ERT2)* embryos followed by a transient incubation in cyclofen. Constitutive induction was achieved by co-injecting *UAS:Cre; ubi:Eos* and *ubi:loxP-Eos-loxP-kRASG12V-T2A-mTFP* plasmids into *Tg(ubi:Gal4-ERT2)* embryos followed by a transient incubation in cyclofen. (A, B) The fluorescent expression of both EosFP and CFP (or mTFP) co-expressed with *kRASG12V* was imaged over 13 days. Quantitative measurements of the fluorescent intensity and qPCR analysis (of *kRASG12V* transcripts) displayed a gradual decrease of *kRASG12V* expression upon transient induction (C, E), and a stable kRASG12V expression upon constitutive induction (D, F). Scale bar: 400 µm. Data are presented as mean ± SEM. ***$p < 0.001$; **$p < 0.01$; *$p < 0.05$; N.S, not significant.

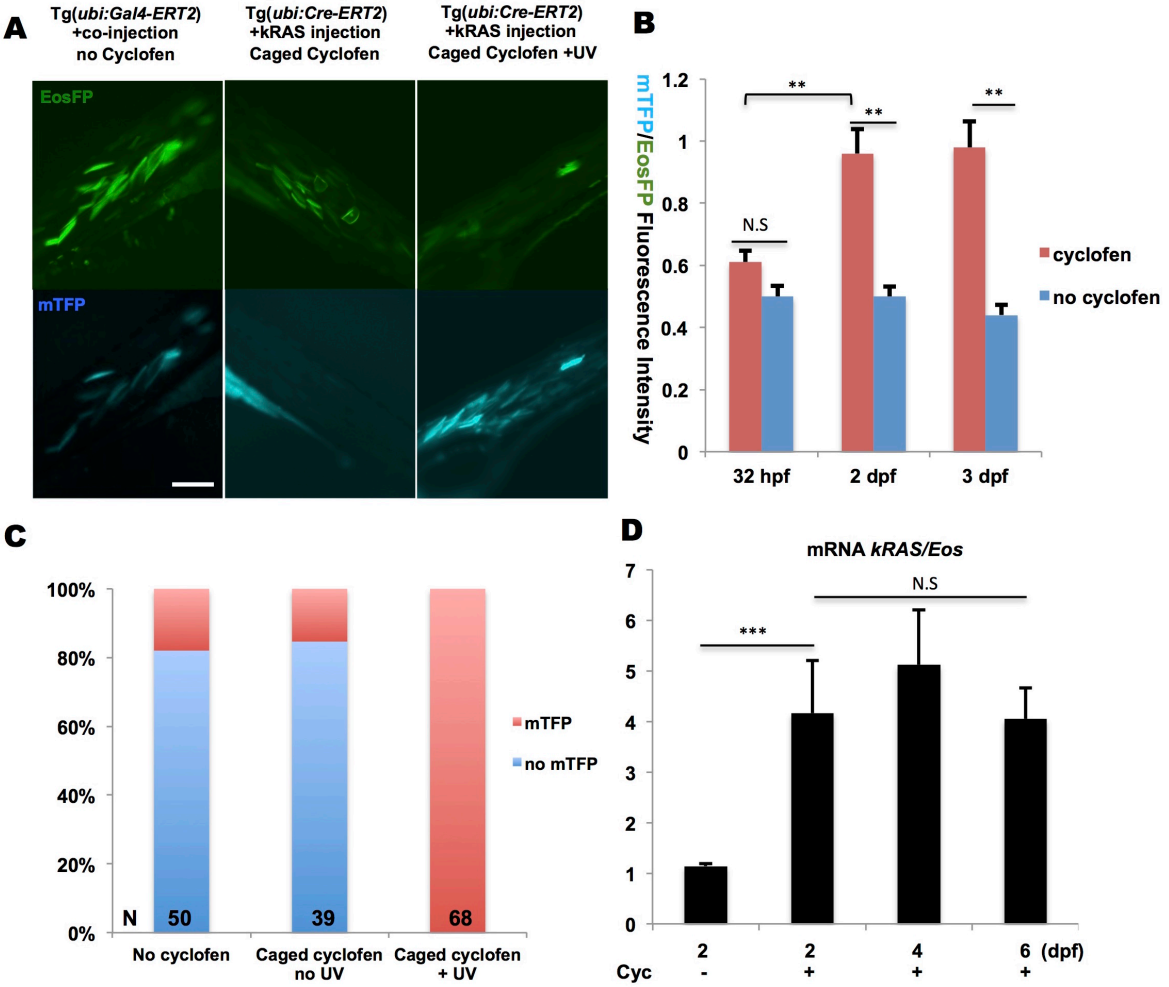

Fig. S2. Optical control of constitutive expression by Cre-recombinase. (A, left panel) *Tg(ubi:Gal4-ERT2)* embryos were injected at the one-cell stage with *UAS:Cre; ubi:Eos* and *ubi:loxP-Eos-loxP-kRAS-T2A-mTFP* plasmids. At 32 hpf, embryos displayed expression of mTFP in spite of absence of cyclofen, indicative of residual activity of Cre-recombinase possibly due to leakage of the UAS promoter. (B) Upon cyclofen treatment at 32 hpf, the expression of mTFP at 2dpf and 3dpf was higher than the control (without cyclofen) . (A, middle panel) Injection of *ubi:loxP-Eos-stop-loxP-kRASG12V-T2A-mTFP* plasmid into *Tg(ubi:Cre-ERT2)* embryos display no leakage in most of the non-activated embryos, as evidenced by the absence of mTFP expression following incubation in caged cyclofen without UV uncaging (despite auto-fluorescence from the yolk). (A, right panel and C) On the other hand, upon UV illumination and release of cyclofen, the *Eos* sequence was effectively excised (resulting in lower fluorescence in the EosFP channel) and the mTFP expression (and fluorescence) was turned on. (D) With *Tg(ubi:Cre-ERT2)* embryos injected with *ubi:loxP-Eos-stop-loxP-kRASG12V-T2A-mTFP* plasmid, RT-qPCR analysis of *Eos* and *kRASG12V* mRNA showed significant increase of *kRAS/Eos* ratio upon cyclofen activation. Scale bar: 200 µm. Data are presented as mean ± SEM. $^{**}p < 0.01$; $^{***}p < 0.001$; N.S, not significant.

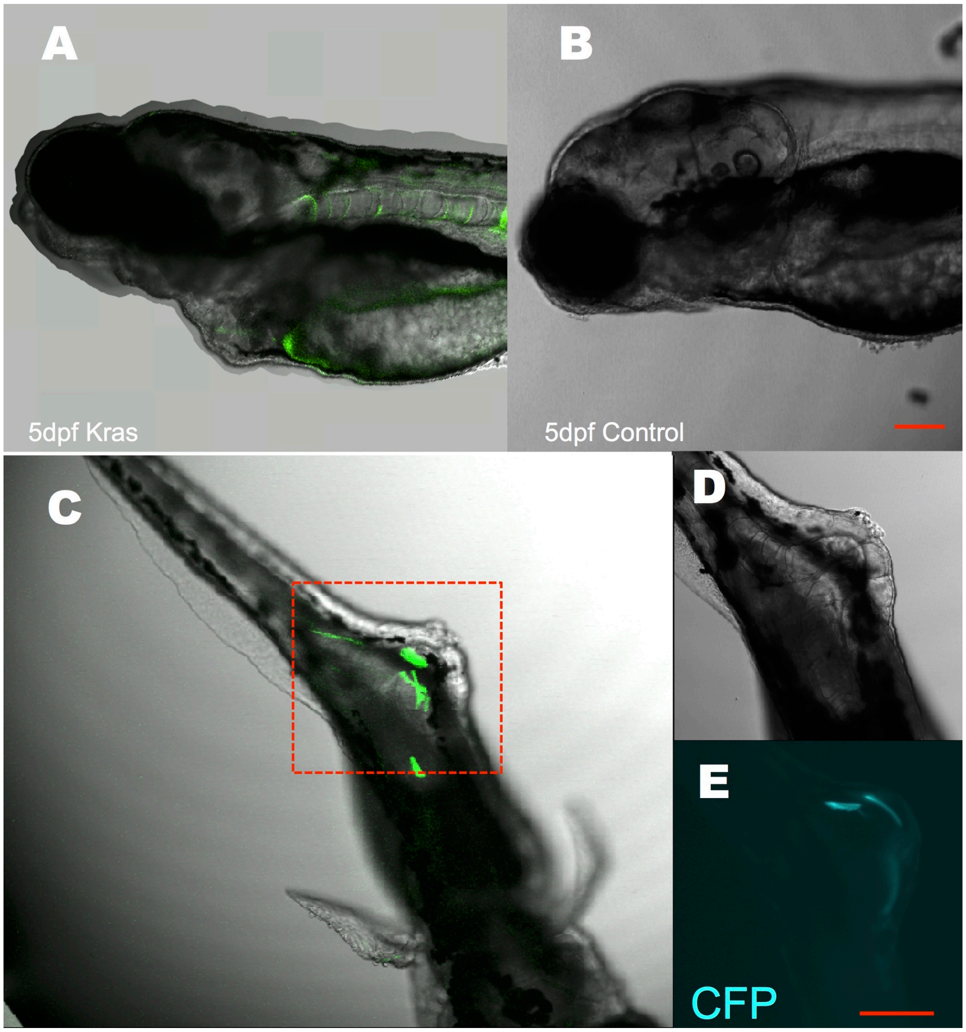

Fig. S3. Transient induction of *kRASG12V* at an early stage resulted in developmental and tumorigenic defects. An early administration of cyclofen at the one-cell stage caused severe developmental defects in 5 dpf fish (A) compared to non-treated control (B). Many fish also exhibited tumorigenic and hyperplasic tissues (C) with remaining CFP expression (D, E zoom-in images of C). Scale bar: 200 µm.

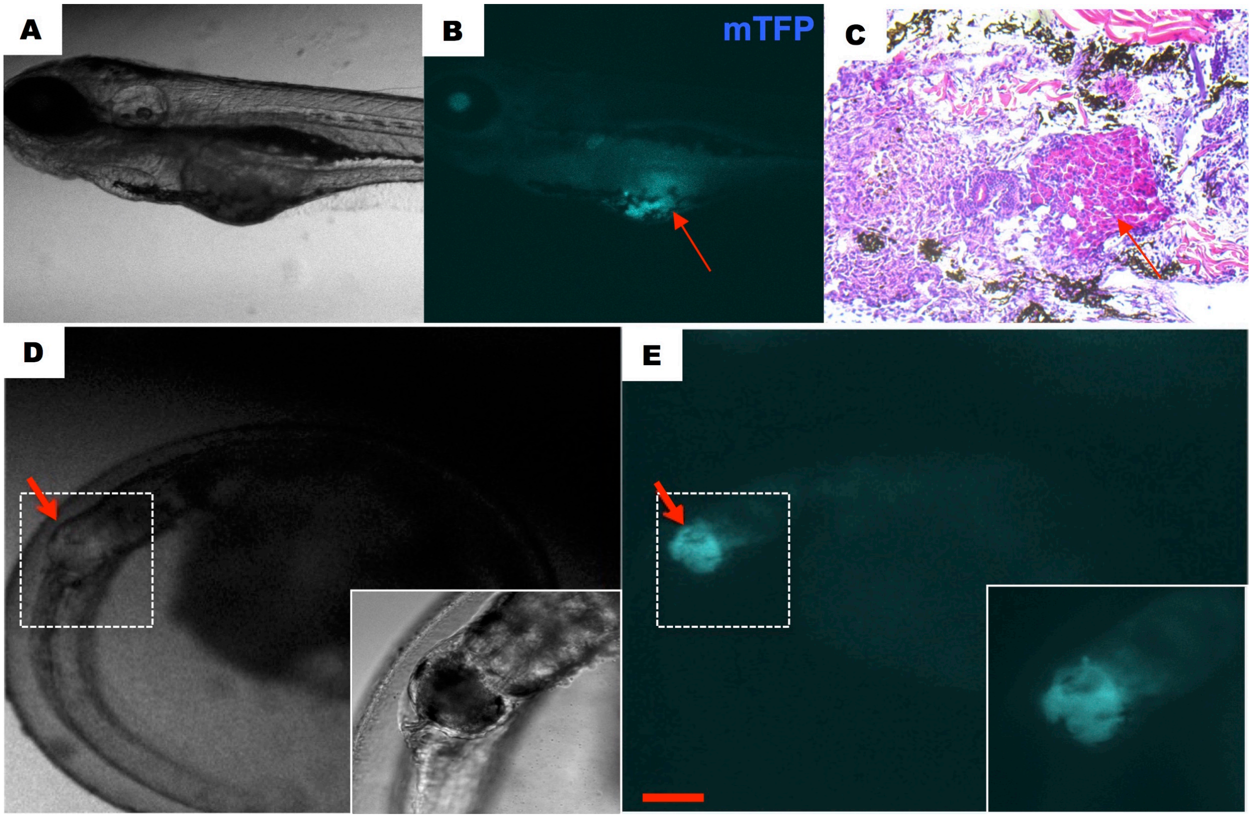

Fig. S4. Early tumorigenesis caused by constitutive activation of *kRASG12V*. Constitutive activation was induced at 1dpf and representative fish showed early tumorigenesis at 5dpf (A-C) and 3dpf (D, E). Notice the hyperplasic tissue (shown by an arrow in B, C), which displayed upon H&E staining a pack of cells with condensed nuclei and an increased nuclei/cytoplasm ratio characteristic of tumors. Scale bar: 200 μm.

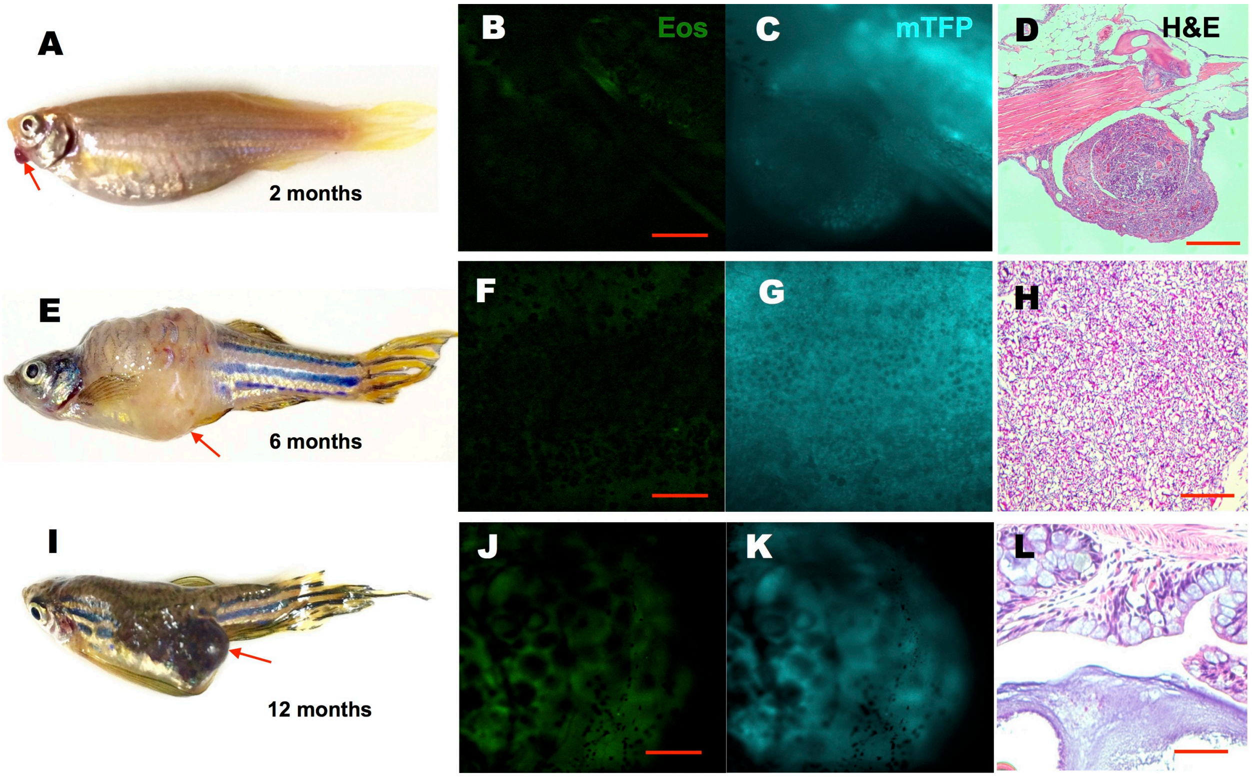

Fig. S5. Representative tumors induced by constitutive *kRASG12V* activation. *Tg(ubi:Cre-ERT2)* fish injected with *ubi:loxP-Eos-stop-loxP-kRASG12V-T2A-mTFP* plasmid were incubated at 1dpf in cyclofen or caged cyclofen + UV. Various tumors (noted by arrows) were observed in fish at 2 (A), 6 (E) and 12 (I) months. The tumors expressed reduced EosFP (B, F, J), and were characterized by both strong expression of mTFP (C, G, K) and histopathological analysis (D, H, L). Condensed nuclei and distorted cell shapes are typical of tumor morphologies, however the different overall textures in D, H and L suggest different tumor types. Scale bar: B, D, F, G, J, 400 µm; D, H 200 µm; L 50 µm.

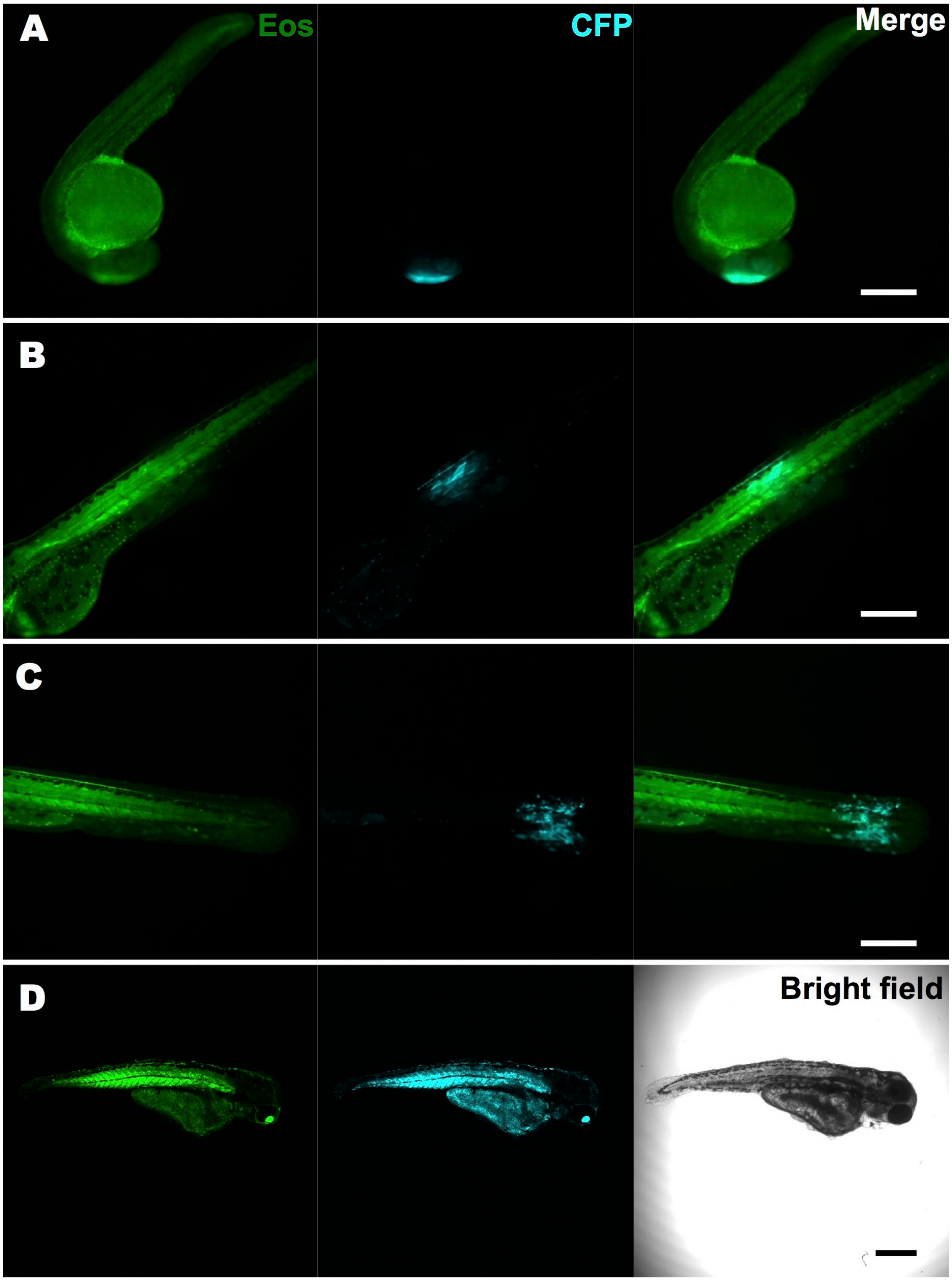

Fig. S6. Local transient activation of oncogene expression with a UV laser at different stages and in various tissues. We crossed a stable *Tg(ubi:Eos;UAS:kRASG12V-T2A-CFP)* line with a *Tg(ubi:Gal4-ERT2)* line. Embryos were pre-incubated in 4 µM caged cyclofen for 4 hours. They were illuminated at 10 hpf (A) or 32 hpf (B, C) with the 405 nm line of a LEICA SP5-BLUE microscope for 5 seconds. Fluorescent images were taken after 18 hours and showed precise spatial control of oncogene activation. While all locally illuminated embryos developed normally, many globally illuminated fish (D) exhibited severe developmental defects and died. Scale bar: 400 µm.

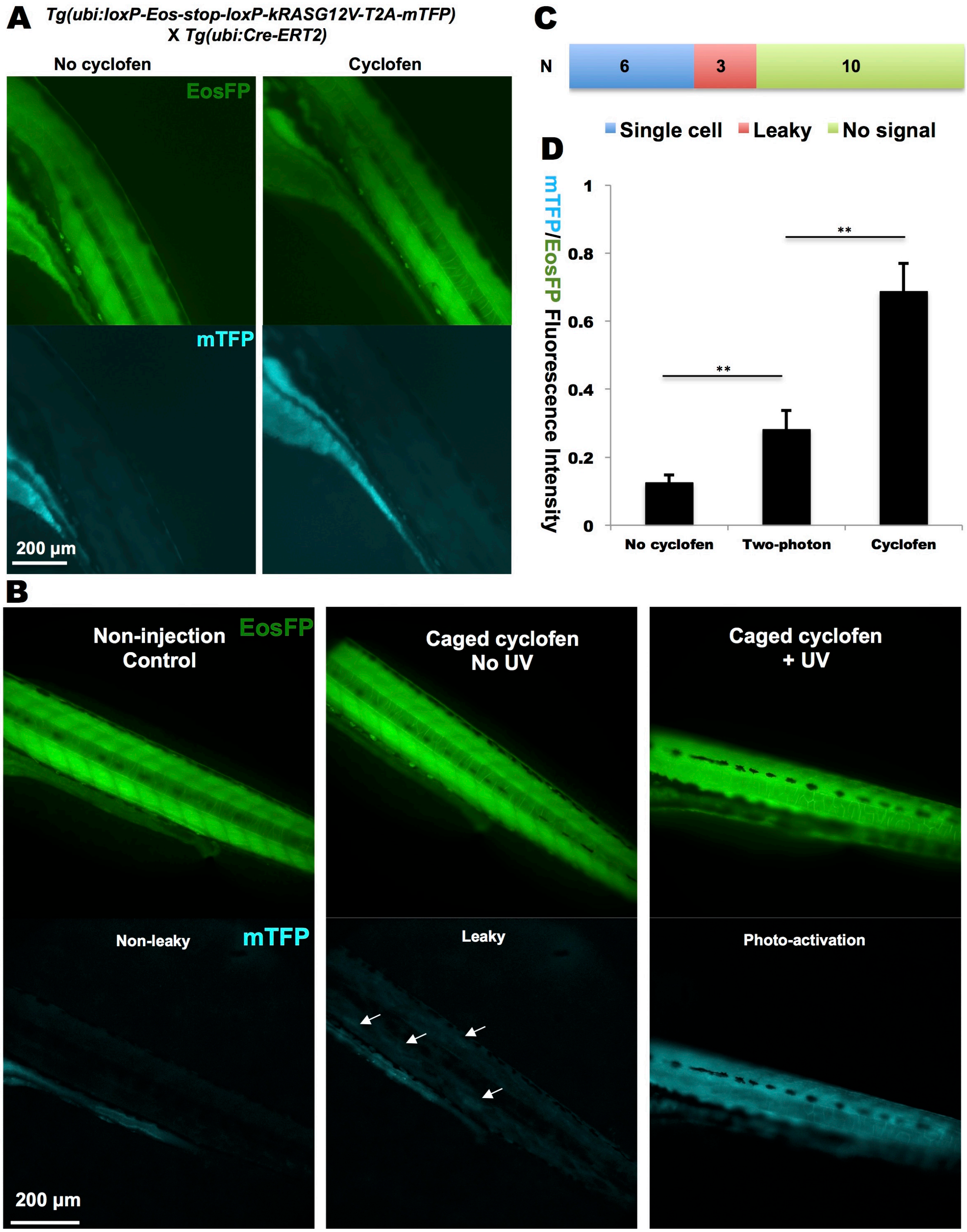

Fig. S7. Constitutive activation of *kRASG12V* in transgenic fish lines. (A) Cyclofen was not able to induce mTFP expression in the heterozygous *Tg(ubi:loxP-Eos-stop-kRASG12V-T2A-mTFP; ubi:Cre-ERT2*) embryo. (B) Oncogene activation could be observed in a *Tg(ubi:loxP-Eos-stop-kRASG12V-T2A-mTFP)* injected with Cre-ERT2 mRNA: distinct mTFP expression patterns in non-activated, leaky (see arrows) and photo-activated embryos are shown. (C)

Success rate of two-photon activation of the oncogene (via the fluorescence of the reporter protein, mTFP) in 19 embryos. (D) Quantification of two-photon activated *kRASG12V* signal compared to non-activated and cyclofen-activated mTFP/EosFP fluorescent ratios.
Data are presented as mean ± SEM. $^{**}p < 0.01$.

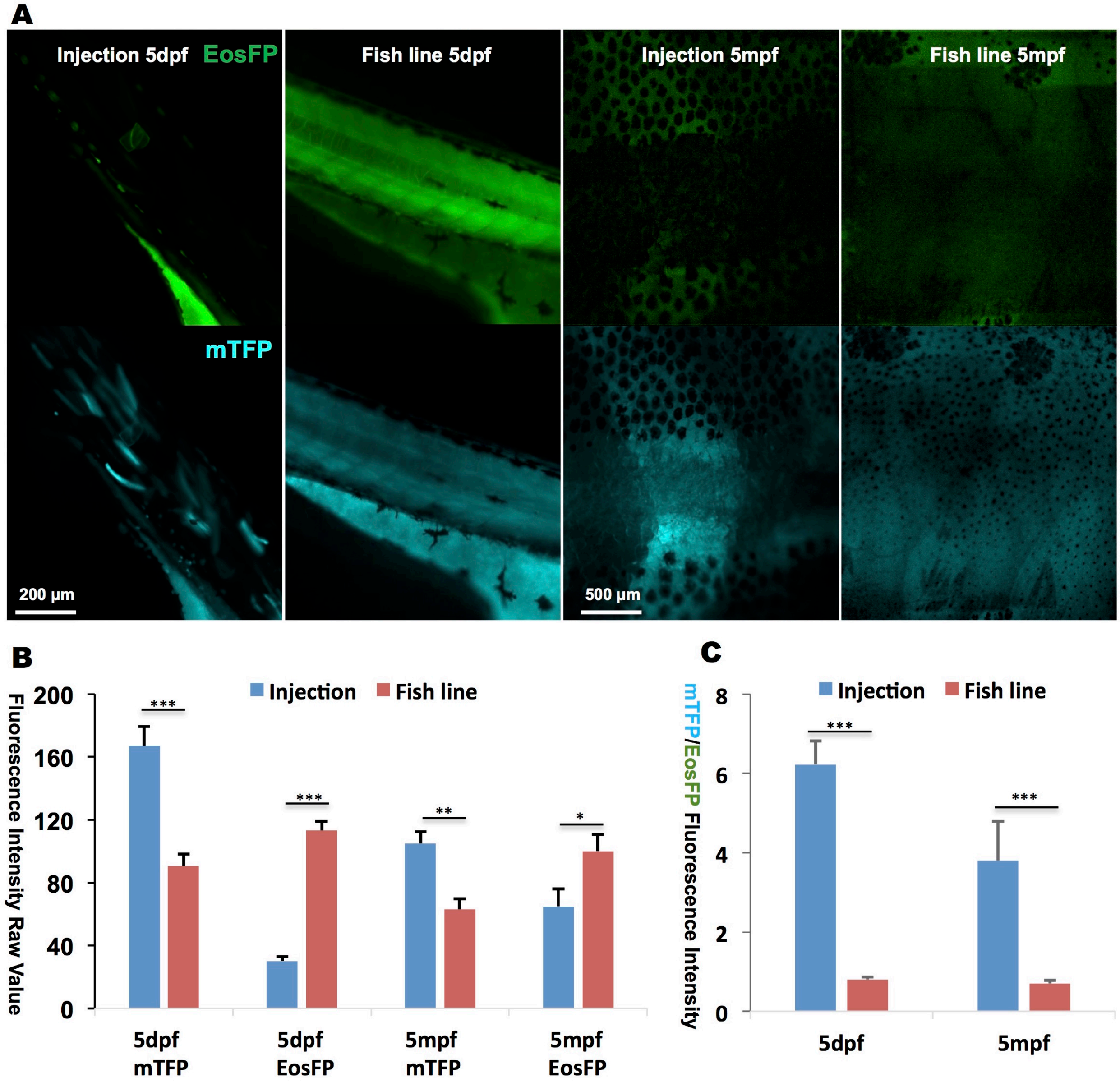

Fig. S8. Lower expression levels of mTFP are induced in the transgenic line as compared to the injected fish. (A) Global photoactivation with a UV lamp of caged cyclofen resulted in higher mTFP fluorescence (and lower Eos fluorescence) in the injected fish than in the transgenic line at both 5 dpf and 5 mpf, measured by both mTFP and EosFP raw intensity (B) and mTFP/EosFP ratio (C). Data are presented as mean ± SEM. $^{**}p < 0.01$; $^{***}p < 0.001$.
